# Comparative analysis and supragenome modeling of twelve *Moraxella catarrhalis *clinical isolates

**DOI:** 10.1186/1471-2164-12-70

**Published:** 2011-01-26

**Authors:** Jeremiah J Davie, Josh Earl, Stefan PW de Vries, Azad Ahmed, Fen Z Hu, Hester J Bootsma, Kim Stol, Peter WM Hermans, Robert M Wadowsky, Garth D Ehrlich, John P Hays, Anthony A Campagnari

**Affiliations:** 1 Department of Microbiology and Immunology, University at Buffalo, Buffalo, New York, USA; 2 Department of Medicine, Division of Infectious Disease, University at Buffalo, Buffalo, New York, USA; 3 Witebsky Center for Microbial Pathogenesis and Immunology, University at Buffalo, Buffalo, New York, USA; 4 Center for Genomic Sciences, Allegheny-Singer Research Institute, Pittsburgh, Pennsylvania, USA; 5 Department of Microbiology and Immunology, Drexel University College of Medicine, Allegheny Campus, Pittsburgh, Pennsylvania, USA; 6 Department of Otolaryngology-Head and Neck Surgery, Drexel University College of Medicine, Allegheny Campus, Pittsburgh, Pennsylvania, USA; 7 Laboratory of Pediatric Infectious Diseases, Radboud University Nijmegen Medical Centre, Nijmegen, The Netherlands; 8 Department of Pathology, School of Medicine, Graduate School of Public Health, University of Pittsburgh, Pittsburgh, Pennsylvania, USA; 9 Department of Infectious Diseases and Microbiology, University of Pittsburgh, Pittsburgh, Pennsylvania, USA; 10 Children's Hospital of Pittsburgh, Pittsburgh, Pennsylvania, USA; 11 Department of Medical Microbiology and Infectious Diseases, Erasmus University Medical Centre (Erasmus MC), Rotterdam, The Netherlands

## Abstract

**Background:**

*M. catarrhalis *is a gram-negative, gamma-proteobacterium and an opportunistic human pathogen associated with otitis media (OM) and exacerbations of chronic obstructive pulmonary disease (COPD). With direct and indirect costs for treating these conditions annually exceeding $33 billion in the United States alone, and nearly ubiquitous resistance to beta-lactam antibiotics among *M. catarrhalis *clinical isolates, a greater understanding of this pathogen's genome and its variability among isolates is needed.

**Results:**

The genomic sequences of ten geographically and phenotypically diverse clinical isolates of *M. catarrhalis *were determined and analyzed together with two publicly available genomes. These twelve genomes were subjected to detailed comparative and predictive analyses aimed at characterizing the supragenome and understanding the metabolic and pathogenic potential of this species. A total of 2383 gene clusters were identified, of which 1755 are core with the remaining 628 clusters unevenly distributed among the twelve isolates. These findings are consistent with the distributed genome hypothesis (DGH), which posits that the species genome possesses a far greater number of genes than any single isolate. Multiple and pair-wise whole genome alignments highlight limited chromosomal re-arrangement.

**Conclusions:**

*M. catarrhalis *gene content and chromosomal organization data, although supportive of the DGH, show modest overall genic diversity. These findings are in stark contrast with the reported heterogeneity of the species as a whole, as wells as to other bacterial pathogens mediating OM and COPD, providing important insight into *M. catarrhalis *pathogenesis that will aid in the development of novel therapeutic regimens.

## Background

*Moraxella catarrhalis *are pathogenic, gram-negative diplococci that colonize the mucosal tissues of the human nasopharynx and respiratory tract by forming biofilms [1-4]. *M. catarrhalis *is one of the three primary bacterial pathogens etiologically associated with otitis media (OM), along with non-typeable *Haemophilus influenzae *(NTHi) and *Streptococcus pneumoniae *[1,3]. Instances of OM are responsible for more visits to healthcare providers than any other pediatric disease in the developed world and infections with *M. catarrhalis *cause approximately 20% of all incidences (recently reviewed in [5,6] and [7]). Additionally, OM is a leading cause of preventable hearing loss in children worldwide [7-9], adding greatly to its public health cost. Furthemore, *M. catarrhalis *is the second most common bacterial cause of exacerbations of chronic obstructive pulmonary disease (COPD) after *H. influenzae *[10]. COPD is among the top five causes of death worldwide and infections with *M. catarrhalis *cause 2-4 million exacerbations of COPD per annum in the United States alone [7,11]. Collectively, these respiratory tract infections create a major burden on the healthcare system, accounting for a combined annual loss conservatively estimated at $33 billion in the United States alone [11,12]. From a public health perspective, however, the point of greatest concern regarding OM and COPD is the high frequency of antibiotic administration, leading to the development of multiply resistant isolates [6,7]. Nearly 100% of *M. catarrhalis *clinical isolates are beta-lactam resistant, underscoring the need to develop alternative treatment modalities, including a *M. catarrhalis *vaccine (recently reviewed in [3]).

Recently, de Vries et al published the first complete *M. catarrhalis *genome, strain RH4, and compared it to the unpublished genome sequence of ATCC 43617 [13]. The gene content of these two strains demonstrated a high degree of homology, suggesting *M. catarrhalis *clinical isolates may possess only limited genetic diversity, in contrast to the reported heterogeneity of the species [14-16]. The Distributed Genome Hypothesis holds that pathogenic bacteria, especially those causing chronic infections, have access to a supragenome that is larger than that of any individual member of that species and that, through homologous recombination, individual strains shuffle their genetic information as a defensive response to assault by the host [17]. Consequently, any effort at controlling *M. catarrhalis*, whether via vaccine or chemotherapeutic intervention, must be rooted in a firm understanding of the core and distributed elements of this species genomic composition. Toward that end, we characterized the supragenome of *M. catarrhalis*, determined from the sequencing and comparative genomic analysis of twelve clinical isolates. Furthermore, mathematical modeling demonstrated that we have sequenced a sufficient number of *M. catarrhalis *genomes to have adequately characterized both the core and supragenomes of this pathogen. Finally, we provide a context-rich, detailed analysis of the *M. catarrhalis *supragenome and its implications towards pathogenesis.

## Results

### Descriptive Characteristics of the Finite *M. catarrhalis *Supragenome Model

**Figure 1:**
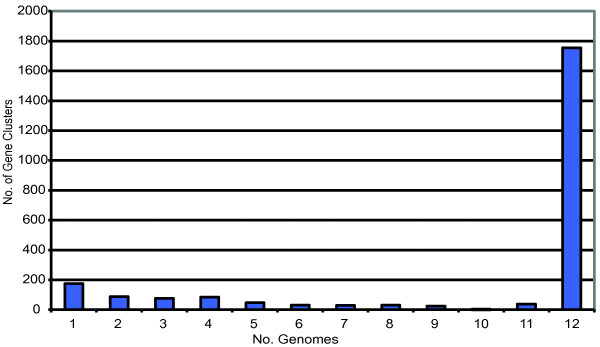
**Distribution of gene clusters among genomes**. The 2383 gene clusters of the *M. catarrhalis *supragenome were plotted according to the number of genomes in which they are present.

**Figure 2:**
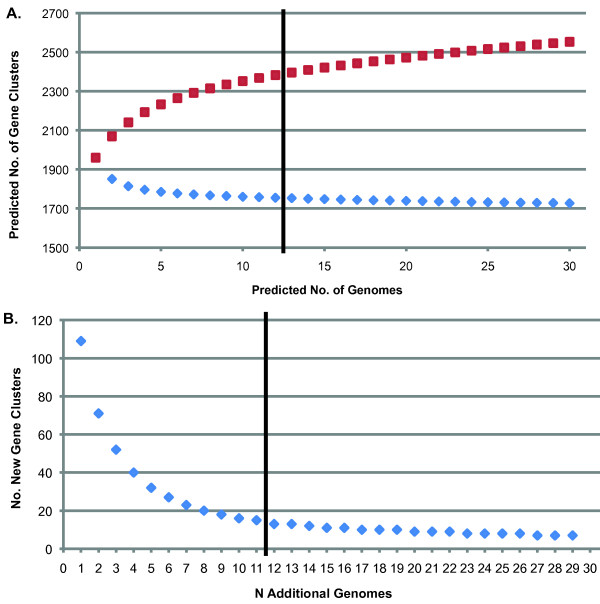
***M. catarrhalis *supragenome predictions**. A) Number of gene clusters in each genome expected by the finite supragenome model. The number of core (diamonds) and total gene clusters (squares) predicted by the finite supragenome model are plotted as a function of *N *genomes. The dividing line identifies the end of values for which we have observed data for comparison and the beginning of the prediction. B) Number of new gene clusters predicted to be identified by the sequencing of additional genomes. Each diamond represents the number of new gene clusters predicted to be identified by the sequencing of one genome and its subsequent addition to *N *genomes present in the model. The dividing line identifies the end of values for which we have observed data for comparison and the beginning of the prediction.

Detailed descriptions of the strains utilized in this analysis are present in Table 1. The strains chosen for this analysis represent a geographically and clinically diverse collection of isolates from the middle ear, respiratory tract and blood stream collected from North America and Europe ([18,19]; this study). *M. catarrhalis *genomes range between 1.78 to 1.96 Mbp (an 10.1% difference), with a mean size of 1.89 Mbp (Table 1). This range represents 95.3% to 105.3% of the previously published *M. catarrhalis *RH4 genome [13]. Interestingly, RH4 is among the smallest sequenced isolates reported to date; only strains O35E, 46P47B1, and 12P80B1 are as small or smaller. The remaining strains possess an average of 57 kbp of additional DNA compared to RH4. The twelve *M. catarrhalis *strains analyzed in this study collectively contain 21,960 predicted coding sequences (CDS). These data were used to predict both the percent coverage of the species supragenome we had achieved, and the total number of genes present in the species using the finite supragenome model as described previously [20,21]. Grouping of the CDS into clusters of orthologous genes identified 2383 clusters; of these, 1755 are present in each strain, representing the core genome of *M. catarrhalis *(Table 2). The remaining 628 gene clusters are either present in multiple, but not all, strains (453) or found only in a single strain (175), and represent the distributed and unique gene clusters of the supragenome, respectively (Figure 1). Figure 2A describes the change in the number of both the core and total gene clusters predicted by the finite supragenome model as each additional genome is sequenced. Likewise, Figure 2B illustrates the expected number of novel gene clusters identified with the inclusion of each additional sequenced genome to the supragenome.

**Table 1:** Strains used in this study.

Strain	Location of Isolation	Clinical Details	Avg. Read Coverage	No. of Contigs	No. Bases in Contigs	% GC	% of RH4 Genome	**NCBI Accession No**.	Source
7169	Buffalo, NY, USA	OME; Child	182.3	35	1,903,901	41.7	102.2	AERC01000000**	Howard Faden
103P14B1	Buffalo, NY, USA	COPD Exacerbation; Adult	19.4	99	1,961,697	41.7	105.3	AERE01000000**	Timothy Murphy
12P80B1	Buffalo, NY, USA	COPD Exacerbation; Adult	19.9	53	1,814,430	41.5	97.4	AERG01000000**	Timothy Murphy
46P47B1	Buffalo, NY, USA	COPD Exacerbation; Adult	20.6	69	1,856,042	41.6	99.6	AERF01000000**	Timothy Murphy
035E	Dallas, TX, USA	OME; Child	56.8	42	1,776,416	41.7	95.3	AERL01000000**	[19]
BC1	Pittsburgh, PA, USA	Tracheal Aspirate, Bronchiolitis; Child	40.2	43	1,954,090	41.4	104.8	AERH01000000**	Robert Wadowsky
BC7	Pittsburgh, PA, USA	Middle Ear, OME; Child	28.7	37	1,904,293	41.5	102.2	AERI01000000**	Robert Wadowsky
BC8	Pittsburgh, PA, USA	Sinus Wash, Sinusitis; Child	43.8	32	1,912,542	41.6	102.6	AERJ01000000**	Robert Wadowsky
CO72	Nijmegen, The Netherlands	Middle ear fluid (OME); Child	37.1	25	1,947,635	41.4	104.5	AERK01000000**	Peter Hermans
101P30B1	Buffalo, NY, USA	COPD Exacerbation; Adult	33.2	26	1,866,092	41.7	100.1	AEPC01000000**	Timothy Murphy
RH4	Denmark	Blood Isolate	Published in [13]	1	1,863,286	41.7	N/A	CP002005***	[18]
ATCC 43617	Belgium	Transtracheal aspirate, chronic bronchitis; Coal miner	NP*	41	1,913,584	41.7	102.7	AX067426*** to AX067466***	ATCC

OME = Otitis media with effusion. * = Not published; the unpublished genome sequence of ATCC 43617 was deposited into GenBank without annotations describing its sequencing methods. ** The accession numbers listed in Table 1 reflect the first version of these whole genome shotgun projects as well as the version current at the time of manuscript submission; master accession numbers for these strains are available in the Methods. *** Denotes original GenBank accession numbers for previously sequenced genomes that were re-annotated for inclusion in this study.

**Table 2:** Comparison of core, contingency and unique gene clusters by genome.

			Contingency				
						
Strain	Genome Size	Core	Distributed *	Unique	Total clusters	%Distributed	%Core
103P14B1	1.96 Mbp	1755	299	24	2054	14.6	85.4
BC1	1.95 Mbp	1755	278	5	2033	13.7	86.3
CO72	1.95 Mbp	1755	274	6	2029	13.5	86.5
ATCC 43617	1.91 Mbp	1755	231	12	1986	11.6	88.4
BC8	1.91 Mbp	1755	223	13	1978	11.3	88.7
7169	1.90 Mbp	1755	224	6	1979	11.3	88.7
BC7	1.90 Mbp	1755	235	86	1990	11.8	88.2
46P47B1	1.86 Mbp	1755	187	18	1942	9.6	90.4
101P30B1	1.87 Mbp	1755	185	1	1940	9.5	90.5
O35E	1.78 Mbp	1755	67	0	1822	3.7	96.3
RH4	1.86 Mbp	1755	147	4	1902	7.7	92.3
12P80B1	1.81 Mbp	1755	120	2	1875	6.4	93.6

* denotes the inclusion of unique clusters within the distributed category.

### Relationship Analysis of *M. catarrhalis *Genome Isolates

**Figure 3:**
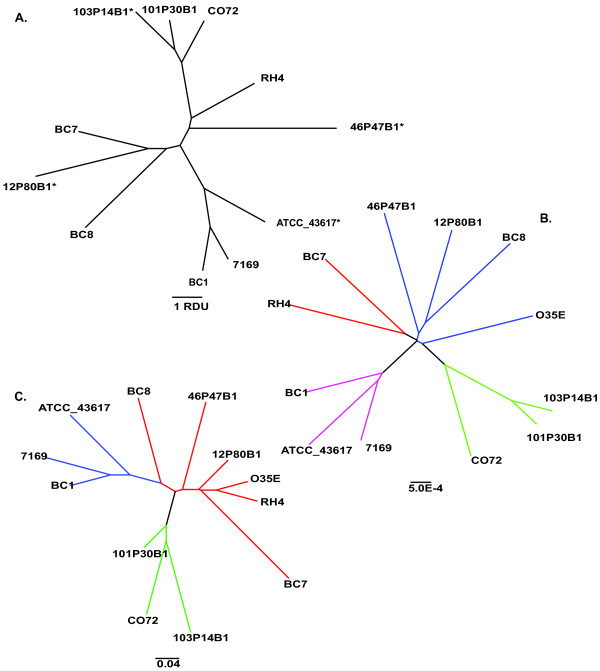
**Relatedness among strains**. Each strain was assayed for its relatedness to every other strain by several methods. A) The MLST type of each isolate was identified as described in the Methods; the scale bar indicates topological distance in relative distance units (RDU) between strains. Isolate O35E was excluded from A) as it contained two sequence variants (*adk *and *fumC *alleles) that were not present in the *M. catarrhalis *MLST database at the time of submission. In addition, * denotes strains whose MLST types were taken from the *M. catarrhalis *MLST database [16]; all other data presented here are taken from pyrosequencing efforts. Neighbor-Joining analysis of was performed for both point mutations in the core genome (B) or genic differences in the distributed (C) genome. Branches in the same neighbor group are identically color-coded and length bars indicate the topological distance measured in 1-average nucleotide identity between each genome.

MLST analyses demonstrated that each of the twelve genomes represented a different sequence type, including four novel sequence types and novel sequence variants for the *fumC *and *adk *alleles present in O35E (Table 3 and Figure 3A), indicating success in selecting a diverse group of strains for sequence analysis. Because of the expected high degree of genomic mosaicism resulting from extensive horizontal gene transfer, relationship dendrograms were constructed by employing a non-phylogenetic identity-by-state, whole-genome clustering method [14,22]. In one case, sequence polymorphisms present in the core genes were used to construct the relationships, and in a second analysis gene possession data were used (Figures 3B and 3C, respectively). These analyses revealed intriguing differences depending upon the analytical method; no clear clade structure could be determined using allelic differences within the core genome, however, the use of the distributed genome data produced a dendrogram that was broadly similar to that generated from the MLST data, albeit with several exceptions. Interestingly, all three methods consistently formed two clusters; one cluster consisting of strains ATCC 43617, 7169, and BC1, and a second group comprised of 101P30B1, CO72, and 103P14B1. Notably, the consistent clustering of these two groups of strains does not correlate with patient age group, geographic origin, COPD exacerbation or OM. These data suggest that using any of these methods alone is insufficient for correctly inferring the relationships among *M. catarrhalis *isolates.

**Table 3:** MLST sequence variants by genome.

	Sequence Variant								
		
Strain	*abcZ*	*adk*	*efp*	*fumC*	*glyBETA (RS)*	*mutY*	*ppa*	*trpE*	Sequence Type
12P80B1*	50	25	12	3	37	52	3	2	185
103P14B1*	8	26	2	3	2	22	41	2	187
ATCC 43617*	3	3	3	4	18	3	3	2	25
46P47B1*	3	20	2	7	62	15	8	2	186
7169	3	18	3	4	3	9	3	2	82
BC1	25	18	3	4	6	9	3	2	NP-ST
BC7	8	18	12	3	29	3	3	2	NP-ST
BC8	2	2	12	7	20	6	3	2	162
CO72	8	8	2	3	2	3	9	2	199
101P30B1	8	26	2	3	2	3	25	2	NP-ST
035E	2	NP-SV	2	NP-SV	57	22	8	2	NP-ST
RH4*	8	30	2	7	32	3	3	2	128

Unless previously determined (*), the eight genes used for the *M. catarrhalis *MLST typing scheme were identified within each genome and used to query the MLST database for the corresponding sequence variant number [13,16]. The sequence number combinations were then used to identify the sequence type, where NP-ST and NP-SV denote sequence types and sequence variants not present in the MLST database at the time of analysis, respectively.

**Figure 4:**
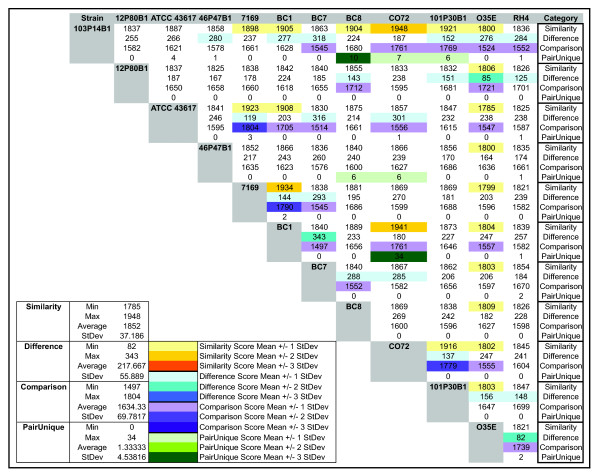
**Pair-wise comparisons of the gene content of the 12 *M. catarrhalis *strains used in this study**. Strain vs. strain comparisons are characterized as described in the text. Values in which one strain differs from its partner by one or more standard deviations are denoted by color code.

An exhaustive pair-wise comparison of all possible strain pairs (n = 66) was performed to gauge the mean and range of gene possession variability within the species (Figure 4). This analysis compares the sum of all gene clusters present in both strains (similarity score), the sum of all clusters not present in both strains (difference score) and the remainder of the latter subtracted from the former (comparison score) to provide an objective metric to quantify the relationship between any two strains. These analyses revealed that any two strains differed on average by the possession of 217.7 ± 55.9 gene clusters.

### Analyses for Mobile Genetic Elements and Codon Bias

Searches were performed to identify known mobile genetic elements by querying the annotation records of each genome for plasmid, transposon or phage-associated genes (Table 4). Plasmid-specific sequences were identified in a single genome (BC7), underscoring the paucity of naturally occurring plasmids reported in the *M. catarrhalis *literature ([23,24] and reviewed in [25]).

**Table 4:** Analysis of mobile genetic elements.

Strain	Genome Size	No. CDS	No. Phage ORFs	% Phage	Plasmid ORFs	% Plasmid	Tn ORFs	% Tn
103P14B1	1.96 Mbp	1944	49	2.5	0	0	3	0.15
BC1	1.95 Mbp	1892	19	1.0	0	0	6	0.32
CO72	1.95 Mbp	1875	20	1.1	0	0	3	0.16
ATCC								
43617	1.91 Mbp	1862	26	1.4	0	0	6	0.32
BC8	1.91 Mbp	1844	19	1.0	0	0	7	0.38
7169	1.90 Mbp	1850	28	1.5	0	0	6	0.32
BC7	1.90 Mbp	1859	9	0.5	12	0.6	7	0.38
46P47B1	1.86 Mbp	1818	21	1.2	0	0	8	0.44
101P30B1	1.87 Mbp	1793	22	1.2	0	0	3	0.17
RH4	1.86 Mbp	1777	11	0.6	0	0	7	0.39
O35E	1.78 Mbp	1720	3	0.2	0	0	6	0.35
12P80B1	1.81 Mbp	1726	11	0.6	0	0	7	0.41

Tn = transposon

Interestingly, the BC7 plasmid-associated contig sequence possesses homologs of the bacteriocin/immunity factor complex first described in the *M. catarrhalis *plasmid, pLQ510 [24,26]; however, annotation records for this region also identify an incomplete VirB-family type four secretion system (T4SS), multiple transposases and a resolvase not reported in pLQ510, suggesting that this sequence may represent either an extra-chromosomally maintained conjugative plasmid or an integrative and conjugative element (ICE; recently reviewed in [27]). Phage-associated annotations varied between strains, but comprised only 0.2% to 2.5% of all identified ORFs per genome, while transposon-associated annotations accounted for only 0.15% to 0.44% of all ORFs per genome. Each genome contains at least one homolog of the IS4, IS200 and IS1016 transposon families or sub-families, while five genomes contain an additional IS605 family transposon. Interestingly, an IS1016 element was found adjacent to a nitrate uptake locus in all twelve strains and, in those strains that contained a second IS1016 element, this element was often inserted in near a collection of dehydrogenases and hypothetical proteins. Likewise, the IS4 element was consistently flanked by a predicted aspartate kinase and amino acid/peptide transporter and, in strains with an additional IS4 element, could also be found in close proximity to the IS1016 element located within the nitrate uptake locus. The IS200 element was also found within a locus containing the mechanosensitive ion channel, *mscS*, and the CRISPR-associated protein, NE0113. IS605 elements were found nearby an IS200 element in all cases. These data illustrate a substantial conservation of transposon elements among *M. catarrhalis *clinical isolates.

**Figure 5:**
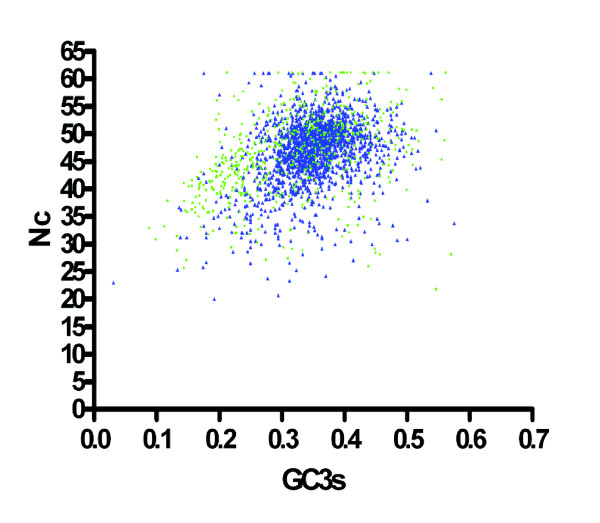
**Comparison of GC3 s plotted against Nc**. Representative sequences from each cluster present in the core (blue triangles) and distributed genomes (green squares) were graphed following the of removal of clusters whose sequences were incompatible with the analysis as described in the Methods.

Genes stably maintained within a species tend to exhibit similar codon usage, whereas recently acquired genes often use less frequent or rarely utilized codons (reviewed in [28]). This difference in codon usage may result from selective pressure towards optimal translational efficiency in each organism, shaped by differences in the iso-accepting tRNA pools available to the donor and recipient bacterial species [29-31]. To this end, a comprehensive analysis of codon usage was undertaken. The total codon usage of the supragenome demonstrates that leucine and cysteine are the most and least encoded amino acids, respectively, while five amino acids (Asn, Lys, Val, Ala, and Gly) demonstrated minor alterations in usage within proteins encoded by the core and distributed genomes (data not shown). A plot of GC usage in the synonymously variable third position (GC3s) against the effective number of codons (Nc) encoded for in each gene present in the core and distributed genome subsets identified a spherical distribution approximately centered at 35% GC3 s for the core genes, whereas the distributed genes were more diffuse, with no central location (Figure 5). These data highlight a preference in the core genome for codons with adenosine or thymidine in the synonymously variable third position, as >80% of core sequences have a GC3 s between 30 and 45%. Conversely, no such bias appears among the distributed genome.

An organism demonstrating an absolute bias toward optimal translational efficiency would utilize a single codon per amino acid in each gene (Nc = 20), whereas an organism displaying no codon usage bias would utilize each codon equally for any given amino acid in a gene (Nc = 61; [32]). As relatively few genes are comprised solely of optimal codons, a gene suspected of exhibiting a translational efficiency bias would be expected to have an Nc score less than 40 [32,33]. The observed mean Nc values for the core and distributed genome subsets are 46.9 ± 5.9 and 46.1 ± 7.4, respectively. However, closer inspection reveals that only 10% (126/1668) of the core genes subset has an Nc value < 40, whereas 18% (170/573) of the distributed genes subset fulfill this criterion, highlighting another difference in codon usage between core and distributed genes and supporting a possible role for translational efficiency in shaping the codon bias of the distributed genome.

### Conservation of CRISPR Elements and Loci Among *M. catarrhalis *Isolates

**Figure 6:**
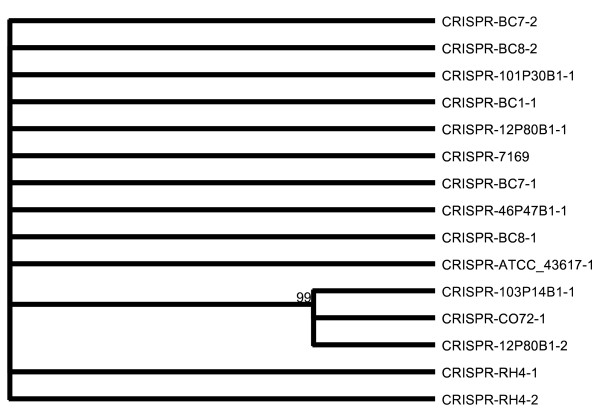
***M. catarrhalis *CRISPR direct repeat consensus sequences cluster into two clades**. The relationship of the direct repeat (DR) consensus sequences of the 15 identified CRISPR loci were analyzed by the Neighbor-Joining method with distances calculated from the absolute number of differences with proportionally distributed gaps, computed from 1000 boot-strapping replications with random tie-braking.

Clustered regularly interspaced short palindromic repeat (CRISPR) elements serve as a means of bacterial self-defense against bacteriophage and plasmid infection (recently reviewed in [34] and [35]). An average prokaryotic genome contains one CRISPR element comprised of 28 repeat-spacer units. The genome of *M. catarrhalis *RH4 possesses a greater than average number of CRISPR loci, containing one putative and two known CRISPR elements [13]. We sought to determine if elevated numbers of CRISPR elements are common amongst isolates of *M. catarrhalis *by analyzing each genome for the number, placement and context of CRISPR loci. Of the twelve strains, only O35E did not contain a CRISPR element. The remaining eleven strains contained an average of 1.4 ± 0.5 CRISPR elements (Table 5). These elements were comprised of direct repeats of 28.1 ± 0.3 nucleotides separated by spacer sequences 32 ± 0.7 bp in length, consistent with previous reports [36,37]. Additional analysis of the direct repeat segments indicated that the direct repeat sequence consensus for each CRISPR element could be split into one of two clades, of which clade 1 contains 80% of the repeat sequences (12/15; Figure 6). While the length of the direct repeat and spacer sequence elements demonstrated only minor variation between strains, the number of spacer sequences varied substantially, ranging between 3 to 48 per element (18.3 ± 11.7 bp). No similarity could be found amongst the spacer consensus sequences between strains, nor does similarity exist between the spacer consensus sequences and a non-*M. catarrhalis *nucleic acid sequence, presently precluding the identification of the invading nucleic acid(s) these sequences defend against.

**Table 5:** Analysis of CRISPR content by genome*.

**Strain**	**BC1**	**BC7**		**BC8**		**CO72**	**101P30B1**	**103P14B1**	**12P80B1**		**46P47B1**	**RH4**		**7169**	**ATCC 43617**
	
**CRISPR Element No**.	1	1	2	1	2	1	1	1	1	2	1	1	2	1	1
**No. Spacers**	24	26	22	9	16	3	15	14	6	22	30	3	48	14	23
**Avg. Spacer Length**	31.9	33.6	31.8	32.2	33.3	31.0	32.1	32.1	32.2	34.0	32.1	32.3	32.0	32.0	32.0
**Direct Repeat Length**	28	28	28	28	28	29	28	28	28	28	28	28	28	28	28
**No. Direct Repeats**	25	27	23	10	17	4	16	15	7	23	31	4	49	15	24

* Isolate O35E is not present in this analysis, as no CRISPR elements were identified in this genome.

Analysis of the gene content flanking the CRISPR elements revealed that half were flanked by CRISPR-associated (Cas) genes. However, a review of the CRISPR position on the draft genome contigs showed several CRISPR regions were present at contig ends, suggesting that the flanking Cas genes may be present in other contig sequences. Concordantly, the genome annotation data identified Cas genes in all genomes, including O35E. Analysis of the surrounding non-Cas genes revealed that CRISPR elements were often located in close proximity to DNA recombination and repair genes. For the eleven strains with confirmed CRISPR elements, the percentage of phage-associated sequence per genome displayed a moderate inverse correlation (*r *= -0.66) with the number of CRISPR elements.

### Whole Genome Comparison of *M. catarrhalis *Isolates and Analysis of Homologous Recombination

**Figure 7:**
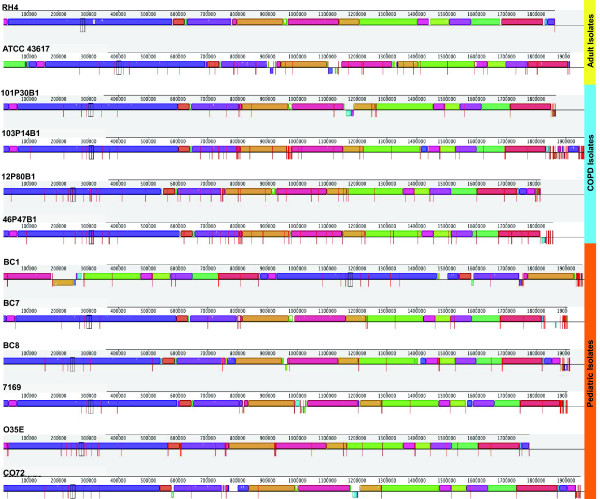
**Whole genome multiple alignment of twelve isolates of *M. catarrhalis***. Genome sequences were categorized by patient group and each homologous LCB is identically colored in each genome. LCB's oriented above the center line for each alignment are in the same orientation as RH4, whereas LCBs oriented below the line indicates an inversion. Unconnected red lines denote contig breaks present in the eleven unfinished genomes.

**Figure 8:**
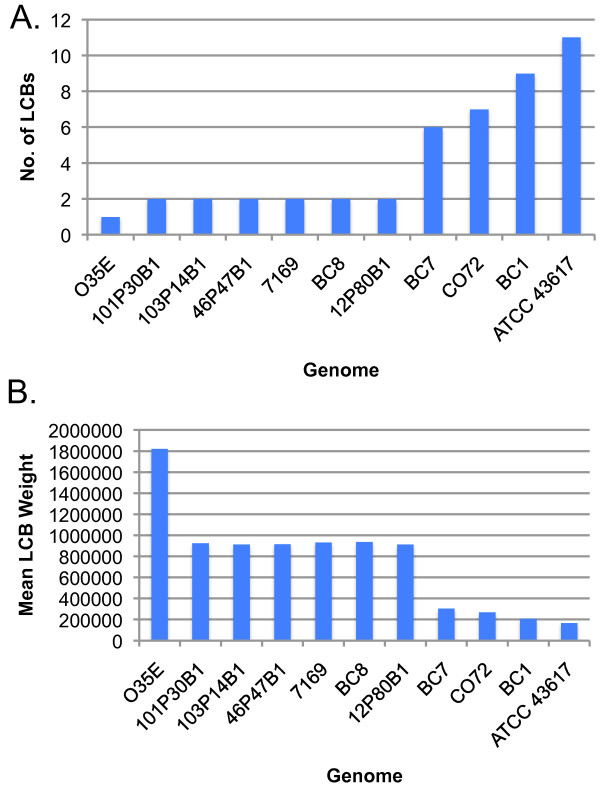
**Comparison of pair-wise alignments of the eleven draft genomes to RH4**. Data are plotted as the (A) number of LCBs common between each labeled genome and RH4, as well as (B) the mean of the LCB weights (as defined in [38]) present in (A).

To facilitate an overview of genetic re-arrangements at the chromosome level, the individual contigs of the 10 *M. catarrhalis *genomes sequenced for this study, as well as the publically available ATCC 43617 draft genome, were ordered relative to the completed RH4 genome. These eleven reordered pseudo-molecules where then compared to RH4 in both whole genome multiple and pair-wise alignments using Mauve [38]. Mauve identifies discrete regions of local homology between genomes that have no internal re-arrangements, known as local collinear blocks (LCBs). The multiple alignment results indicate a high degree of structural conservation, as the alignment is primarily comprised of a small number of large LCBs common to multiple genomes (Figure 7). The overall similarity of LCB placement among the genomes suggested that one or more genomes might be considerably more divergent from RH4 than the majority. Indeed, pair-wise comparisons of each genome to RH4 indicated that the majority of the strains exhibited a high degree of chromosomal synteny with RH4, often comprising the entire genome in 1-4 LCBs (Figure 8). ATCC 43617 differs the most from RH4 (11 LCBs), whereas O35E is nearly identical (1 LCB).

### Metabolic Reconstruction of the *M. catarrhalis *Supragenome

A detailed comparative analysis of the central metabolic pathways of the twelve sequenced *M. catarrhalis *strains was performed (Additional File 1, Table S1). In general, CDS of the enzymes of the central metabolic pathways were highly conserved among all *M. catarrhalis *strains, with only a few instances of apparent frameshift-disrupted ORFs. In some cases, shorter CDSs were identified in one genome, e.g. phosphoglycerate kinase in the ATCC 43617 genome, but none of the genes were completely absent in any genome.

**Figure 9:**
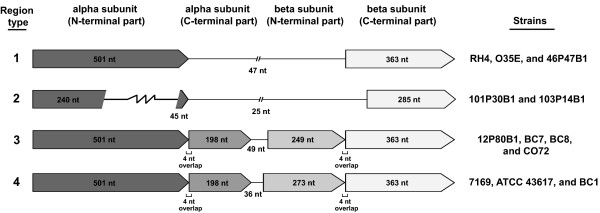
**Overview of variant regions encoding putative succinyl-CoA:3-oxo-acid CoA-transferase (SCOT) subunits in *M. catarrhalis *strains**. Four variant regions were identified: type-1 regions contain two ORFs encoding the N-terminal part of the alpha subunit and the C-terminal part of the beta subunit; type-2 regions hold the same ORFs but one with an internal deletion and one in a truncated form; type-3 regions have two sets of overlapping ORFs encoding the N-terminal (501 nt) and C-terminal part (198 nt) of the alpha subunit and the N-terminal (249 nt) or the C-terminal part (363 nt) of the beta subunit, respectively; type-4 regions are identical to type-3 regions except that the ORF encoding the N-terminal part of the beta subunit is 273 nt in length.

Similar to RH4, all sequenced *M. catarrhalis *genomes appear to possess an incomplete glycolysis pathway, lacking the genes encoding the key enzymes phosphofructokinase and pyruvate kinase, while the gluconeogenic pathway was intact in all analyzed genomes. The tricarboxylic acid (TCA) cycle, while incomplete in *M. catarrhalis *due to the absence of genes encoding both succinyl-Coenzyme A (CoA) synthetase subunits, is otherwise conserved in each genome and this disruption may be bypassed by the conserved glyoxylate shunt. The reversible conversion of succinyl-CoA to succinate by succinyl-CoA synthetase may be substituted by succinyl-CoA:3-oxo-acid CoA-transferase (SCOT, 2.8.3.5), which catalyzes the reversible transfer of Coenzyme A (CoA) from one carboxylic acid to another, e.g. transfer of CoA from succinyl-CoA to oxaloacetate [39,40]. Two ORFs previously annotated in RH4 as putative 3-oxoacid CoA-transferase alpha subunit (MCR_0737) and putative beta subunit (MCR_0738) could potentially catalyze this reaction [13]. However, MCR_0737 only encodes the N-terminal part of the alpha subunit whereas MCR_0738 only encodes the C-terminal part of the beta subunit. Interestingly, a detailed comparative analysis of the corresponding chromosomal region in all strains revealed four variant regions encoding the putative SCOT subunits (Figure 9). Two strains, O35E and 46P47B1 (type 1 region), showed the same organization as RH4, containing two CDS encoding the N-terminal part of the alpha subunit and the C-terminal part of the beta subunit, respectively. Strains 101P30B1 and 103P14B1 (type 2 region) harbored the same genes, but the MCR_0737-ortholog contained an internal deletion, and the MCR_0738-ortholog was found to be truncated. The type 3 and 4 regions contained overlapping ORFs that could encode the missing C-terminal part of the alpha subunit and the missing N-terminal part of the beta subunit, with a small size-difference in the latter (Figure 9). The TCA cycle requires the supply of acetyl-CoA, which may be derived from the degradation of fatty acids and assimilation of acetate, for which the required CDS were identified in all strains. Finally, as in RH4, genes encoding the enzymes of the non-oxidative branch of the pentose-phosphate shunt were present in all strains.

### Distribution of *M. catarrhalis *Virulence Factors

A number of virulence factors have been described in *M. catarrhalis*, ranging from cell-to-cell adhesins (Hag; UspA1/2/2H; McaP; MchA1/A2) and inflammatory mediators (LOS) to biofilm formation (type four pilus (TFP); Recently reviewed in [41,6] and [3]). Each of the genomes was assessed for the presence of known or putative virulence factors (Additional File 2, Table S2). Interestingly, with few exceptions, genes encoding all known virulence factors were present in each strain. While the presence of *uspA2 *or *uspA2 H *is mutually exclusive, the extreme skew in genomes containing *uspA2 H *is inconsistent with previous reports and would appear to be the result of an automatic assembly and/or annotation bias resulting from the repetitive sequence cassettes that comprise these genes [42,43]. In addition, the presence of *mchA1/A2 *is also considerably more varied than the literature would suggest; however, the high degree of sequence homology shared between these two adhesins makes an absolute accounting of their presence challenging without a targeted sequencing effort (see Discussion; [44,45]).

## Discussion

In the present study, we report genome assemblies for 10 *M. catarrhalis *clinical isolates that exceed the criteria established by the Human Microbiome Project for draft genome sequences and illustrate that the sequencing of each genome is practically complete [46]. Comparative genomic analysis has revealed 2,383 orthologous gene clusters, 74% of which are present in all strains (Table 2). The remaining 26% are found among the twelve genomes in an uneven distribution; 67% of the 628 distributed gene clusters are found in four or fewer genomes (Figure 1). Figure 2 demonstrates that the core genome size is unlikely to decrease substantially with the sequencing of additional genomes; hence the *M. catarrhalis *core has been essentially characterized. Moreover, the finite supragenome model predicts that >85% of the *M. catarrhalis *species supragenome is contained within these twelve strains, and that the vast majority of additional genes to be found are likely to be rare genes, present in less than 10% of strains. The uneven nature of the distributed genome serves to highlight the diversity of this segment of the supragenome and is consistent with the distributed genomes of *S. pneumoniae *and the NTHi [20,21]. In contrast with NTHi and *S. pneumoniae*, whose supragenomes are divided almost evenly among the core and distributed genomes, the division of these genomes in *M. catarrhalis *is 3:1, respectively. Hiller et al. suggested that the large size of the distributed genomes in *S. pneumoniae *and NTHi result, at least partially, from their natural competency and ability to survive within the human host in biofilms, forming a close proximity environment in which genetic information could be exchanged [21]. Despite sharing those two phenotypes with *S. pneumoniae *and the NTHi, a given isolate of *M. catarrhalis *shares 74% of its coding potential with every other isolate, suggesting that some other mechanism(s) function to constrain horizontal acquisition of genetic information in this bacterium [1]. Horizontal gene transfer in *H. influenzae *via natural transformation is skewed toward DNA from members of the *Pasteurellaceae *by the use of uptake signal sequences (USS), which have been recently suggested to facilitate the molecular drive of family-specific DNA [47,48]. While *S. pneumoniae *does not possess an USS, it does contain a stress-induced competence system associated with the fratricidal secretion of antibiotics that has been suggested to contribute to the *S. pneumoniae *distributed genome [49]. However, no system analogous to either of these has been reported in *M. catarrhalis*. Instead, the smaller size of the *M. catarrhalis *distributed genome may be the result of genetic frugality; as *M. catarrhalis*, NTHi, and *S. pneumoniae *are commonly present in polymicrobial infections, *M. catarrhalis *does not need to duplicate the coding potential of these bacteria to benefit from the indirect pathogenicity provided by the substantially more diverse supragenomes of these species [20,21,50]. Support for a model of genomic efficiency in *M. catarrhalis *is found in the recent observation that *M. catarrhalis *7169, one of the twelve strains analyzed in this study, responds to autoinducer-2 signal produced by the NTHi strain 86-028NP, substantially increasing its already robust capacity to form a biofilm [4,51]. Armbruster et al. posit that such a relationship with NTHi is reciprocal as *M. catarrhalis *releases substantial amounts of beta-lactamase into the local environment, conferring beta-lactam resistance to other bacteria in close proximity [51-55]. Furthermore, the work of Armbruster et al. is consistent with the observation of Tan et al. that *M. catarrhalis *and NTHi cooperate to avoid clearance by components of the innate immune system [56]. Clearly, further studies are warranted to explore this multispecies interaction at the supragenome and population levels. Interestingly, analysis of the 16 S rRNA sequence of all strains indicated that they are type 1 isolates, which is reported to predominately group within the sero-resistant lineage (data not shown; [14]). Therefore, it is possible the recent divergence of serum-resistant *M. catarrhalis*, estimated at 5 million years ago, may have limited the opportunity to acquire distributed genes in general and virulence-specific factors in particular [16].

*M. catarrhalis *isolates possess a wide array of virulence factors known or suspected of mediating pathogenesis (reviewed in [3]). Interestingly, the vast majority of the known/putative virulence factors of *M. catarrhalis *are present in the core genome. This finding is instructive, as it demonstrates that the large pool of conserved genes in this species includes many of the most promising vaccine candidates. In particular, these core virulence factors include the type four pilus (TFP) and several adhesin-like proteins, both of which are surface exposed [3,57]. In particular, TFP and UspA1 have been demonstrated to be important in biofilm formation ([4] and recently reviewed in [3]). Notably, *mchA1/2 *sequences have been completely sequenced in ATCC 43617, 7169, and O35E, as well as identified by PCR product amplification in 16 additional *M. catarrhalis *isolates [44,45]. Despite this high level of conservation, only eight out of twelve strains in this study contained large partial ORFs homologous to either *mchA1 *or *mchA2*. However, the absence of complete *mchA1/2 *sequences must be carefully interpreted; *mchA1 *and *mchA2 *are 5.4 kb and 5.2 kb long, respectively, with 100% identity over the first 3.6 kb [44,45]. Furthermore, nine of twelve strains in the present study contain the cognate two-partner secretion pore, *mchB*, suggesting that these strains likely contain a functional *mchA1 *and/or *mchA2*. Similar challenges were observed with *uspA2/2 H *sequencing, as automatic annotation resulted in the identification of fragmented sequences corresponding to twelve *uspA2 H *homologs and no *uspA2 *homologs. While expression of *uspA2 *and *uspA2 H *are exclusive of one another, the automatic PGAAP annotation of the O35E and 7169 genomes incorrectly identifies the *uspA2 *sequence fragments as *uspA2 H *[42] . The fragmented disposition of the *uspA2 *sequence appears to have confounded the automated annotation process, likely caused by the *uspA1- *and *uspA2-*homologous regions that characterize the hybrid nature of *uspA2 H *[43]. These long, repetitive sequence features collectively make it difficult for the automated assembly and annotation programs to correctly characterize certain HMW adhesins like *mchA1/2 *and *uspA2/2 H *from medium-read genome sequencing data. It should be noted that the *mchA *genes were first identified in *M. catarrhalis *ATCC 43617, whose unpublished draft genome sequence was determined using longer Sanger-sequencing reads [44,45]. Similarly, *uspA1 *and *uspA2/2 H *sequences were elucidated by traditional sequencing methods [42,43].

The consistency with which virulence factors in particular, and most genes in general, are present within each genome is remarkable. The general absence of plasmid carriage in the sequenced *M. catarrhalis *isolates is instructive and suggests that the processes of natural transformation, phage- and transposon-mediated horizontal gene transfer serve as the primary means of acquiring genic diversity in this species. The number and sequence diversity of CRISPR elements present in these isolates supports this premise and further suggests that, as a species, *M. catarrhalis *is comes into contact with a diverse population of bacteriophages and other invading nucleic acids in the human host. While the deviation in both CRISPR spacer number and spacer consensus sequences suggests that these strains have likely evolved independently of one another in terms of the specific infectious agents and temporal distribution of resistance events, a conclusive statement requires additional analyses. Hansen and colleagues observed in separate studies that the functional bacteriocin/immunity complex identified in pLQ510 is chromosomally maintained in many isolates and the identification of a putative conjugative plasmid/ICE containing this complex in the BC7 genome may represent another means of shuttling genetic information between *M. catarrhalis *isolates [24,26]. It is interesting to note that all genomes possess at least one transposase from each of the IS4, IS200, and IS1016 families that is present in a similar chromosomal context in each strain, while 5 genomes possess an additional IS605 family transposase. IS200 family transposases have been proposed as a sub-group of the larger IS605 family and the most commonly observed DNA re-arrangement mediated by IS200 family transposases are deletion mutations (Reviewed in [58]). Additionally, IS200/605 transposases lack terminal repeats, permitting them to repair the disrupted gene faithfully without gene duplication upon excision, thus limiting the potential for the creation of novel and potentially advantageous ORFs [58,59]. While IS4 elements utilize a similar "cut-and-paste" form of non-replicative transposition, they do possess terminal inverted repeats and can encourage gene duplication upon excision (Reviewed in [60]). While IS1016 family transposons are not found in all *H. influenzae *isolates, they were first described in this species as a novel class of compound transposon facilitating the amplification of a polysaccharide capsule loci [61,62]; given their apparent ubiquitous distribution among *M. catarrhalis *genomes and the intimate relationship between these two bacteria, it is possible that IS1016 originated in *M. catarrhalis *and has only been recently acquired by *H. influenzae*.

It is notable that, while the sequenced *M. catarrhalis *isolates are nearly devoid of plasmid sequences and consistently display the same repertoire of transposons, the distributed and core genomes show evidence of differential codon bias. Such differences are often the result of the acquisition of foreign DNA (reviewed in [28]), but the observations that 1) the distributed genome is predicted to be better suited than the core genome for optimal translation and 2) the limited number of sequences suspected to be of foreign origin present in the *M. catarrhalis *supragenome suggest that a primary source of genic diversity in this species may be the acquisition of novel genetic elements that have arisen by mutation in another strain of *M. catarrhalis*. Hence, the differences in codon usage in the core and distributed genomes may be due to differing rates of molecular evolution as this pathogen continues its maturation. However, we cannot rule out the possibility that the differences in codon usage witnessed in the core and distributed genomes are the result of an unrecognized bias. Further studies investigating the mutation rates of the core and distributed genomes, as well as characterization of the *in vitro *and *in vivo *transcriptomes, of multiple *M. catarrhalis *strains would be useful in characterizing the evolution of this species.

In addition to characterizing the *M. catarrhalis *supragenome, we investigated the possibility that clinical isolates from patients with OM and COPD represented two distinct populations with respect to chromosomal organization and coding potential. As such, we evaluated each strain at the genomic and genic levels. Multiple and pair-wise whole genome alignments indicate that *M. catarrhalis *isolates exhibit a high degree of chromosomal synteny and have undergone only limited large-scale genome re-arrangement as a species (Figure 7). Similarly, while a metabolic reconstruction analysis revealed intriguing differences in the composition of four variant loci encoding putative SCOT enzyme subunits, the lack of additional differences in the central metabolic pathways of these isolates highlights the nearly identical metabolic potential of the twelve analyzed strains (Figure 9 and Additional File 2, Table S2). While the number of gene clusters present in the distributed genome increases as anticipated with concomitant increases in genome size, strains isolated from COPD or OM patients do not trend toward either end of the genome size spectrum. Furthermore, dendrograms generated using sequence data from the core and distributed gene clusters revealed that the use of either set of gene clusters had a demonstrable differential effect on branch placement within each dendrogram (Figure 3). Additionally, as neither dendrogram correlates with MLST type, geographic origin, patient age group, disease type or SCOT loci variant, these data illustrate the potential for a very minute number of sequence differences to exert an undue bias in the grouping of certain strains. This core/non-core-dependent variation in dendrogram topology is in discordance with similar trees created from the core and distributed genomes of NTHi and *S. pneumoniae*, where the use of either data generated similar dendrograms ([20,21]; G. Ehrlich, unpublished). Taken together, the data presented in this study do not support a genomic differentiation between strains isolated from children and adults and moreover suggest that most strains of *M. catarrhalis *would be capable of causing either OM or exacerbations of COPD.

## Conclusions

This study describes the *M. catarrhalis *supragenome and provides an in-depth characterization of twelve genomes, including ten sequenced expressly for this effort. Comparative analyses revealed that all twelve isolates are highly similar within the context of their virulence and metabolic potential, CRISPR and mobile genetic element content, chromosomal synteny and gene content. Interestingly, while the distribution of gene content among strains is consistent with the DGH, the proportion of genes found in the core and distributed genomes differs considerably with that reported for the OM and COPD-associated pathogens *S. pneumoniae *and NTHi. With nearly three-quarters of genes common to all isolates, including the vast majority of virulence-associated genes, *M. catarrhalis *would appear especially suitable to control via vaccination. It is also of substantial interest that these studies do not reveal obvious genomic differences between isolates recovered from COPD and OM patients, suggesting that, should a distinction exist, it would be mediated by an extremely small number of genes and/or mediated by an unrecognized epigenetic mechanism. The data presented in this study significantly increases our knowledge of *M. catarrhalis *biology and these data could be instrumental in the development of novel antimicrobial treatments designed to lessen the substantial individual, social and economic burdens of COPD and OM.

## Methods

### DNA Sequencing

Genomic DNA was isolated from overnight cultures of *M. catarrhalis *strains (Table 1) using the Genomic-tip 500/G kit (Qiagen, Valencia, CA) as per manufacturers instructions. Genome sequencing was performed using a 454 Genome Sequencer (Roche) using either GS-20 or Titanium chemistries. Strain 7169 was sequenced by a combination of 3-kb and 8-kb 454 Titanium paired-end sequencing, while strains 12P80B1, 103P14B1 and 46P47B1 were subjected to 454 GS-20 whole genome shotgun sequencing; all four strains were sequenced by the Infectious Disease & Genomics Core facility at the NYS Center of Excellence in Bioinformatics and Life Sciences (University at Buffalo, SUNY). Strains BC1, BC7, BC8, CO72, 101P30B1 and O35E were sequenced by 454 Titanium whole genome shotgun sequencing at the Center for Genomic Sciences at the Allegheny-Singer Research Institute. All sequencing protocols and chemistries used were performed as per manufacturers instructions.

### Draft Genome Assembly

Genomes were assembled using the Newbler assembly program (v2.0.01.14 or later) and complete genome assembly statistics are present in Table 1. Each genome was sequenced to a high level of coverage (> 19x) and each draft genome was evaluated by the high-quality draft genome assembly criterion established by the Human Microbiome Project consortium [46].

### Accession Numbers and NCBI Project IDs

The genome sequences novel to this study have been deposited at DDBJ/EMBL/GenBank and are denoted by the following master accession numbers: AEPC00000000 (101P30B1), AERC00000000 (7169), AERE00000000 (103P14B1), AERF00000000 (46P47B1), AERG00000000 (12P80B1), AERH00000000 (BC1), AERI00000000 (BC7), AERJ00000000 (BC8), AERK00000000 (CO72) and AERL00000000 (O35E). Accession numbers for RH4 and ATCC 43617, as well as the version described in this manuscript, are listed in Table 1.

### Gene Identification and Gene Clustering

The assembled contig files were submitted to the NCBI's Prokaryotic Genomes Automatic Annotation Pipeline (PGAAP) [63]. Putative gene sequences identified by this system were used in a clustering algorithm implemented at the Center for Genomic Sciences. Each gene's nucleotide sequence was aligned against every other gene nucleotide sequence from all twelve genomes. To account for sequences that were present in the genome, but not identified by the automatic annotation, the amino acid sequence of each gene was aligned against every nucleotide contig from all twelve genomes. Alignments were done using either the threaded Fasta or Tfasty program version 3.6 [64]. Genes were clustered together by comparing alignment scores. If a gene matched at least one other gene in a cluster with 70% identity, over 70% of the smaller gene length, it was added to a cluster. For a more rigorous mathematical reasoning of this threshold choice, see [20].

### Neighbor Grouping Analysis

All genomes in this study were compared using a neighbor grouping analysis, as described [22]. Essentially, after the gene clustering procedure, two sub-sets of gene clusters were identified, a modified core, defined as a gene cluster that had only one member from every strain, and a modified distributed set, defined as a cluster which had fewer than all strains as a member, but greater than just a single strain. Two grouping analyses were done: creating a distance matrix from allelic differences in the modified core set, and a distance matrix based on the genic presence/absence in the modified distributed set. In short, genomes that shared high percent identities between core genes are "closer" or "less distant" from one another in the allelic comparison. Likewise, genomes that shared membership in many of the same distributed gene clusters would also be more closely related. Distance matrices and statistics were computed using a custom perl implementation. Genomes whose distances from each other were less than the total average distance minus the standard error were grouped together. Trees were built from these matrices using the Phylip Neighbor-Grouping software, with default settings [65].

### Finite Supragenome Model

Predictions for the number of genomes required for the percent coverage of total genome size, novel gene clusters added per genome, and number of core gene clusters was computed via the method described in [20]. The only alteration made to the model was to allow gene class population frequencies to vary; previously the values were fixed.

### MLST Analysis

Multi-locus sequence typing was inferred from the best-hit homologs of *glyRS *(glycyl-tRNA synthetase beta subunit), *ppa *(pyrophosphate phospho-hydrolase), *efp *(elongation factor P), *fumC *(fumarate hydratase), *trpE *(anthranilate synthase component I), *mutY *(adenine glycosylase), *adk *(adenylate kinase) and *abcZ *(ATP-binding protein) present in each genome in compliance with the *M. catarrhalis *MLST scheme developed previously [16]. Each homolog was used to query the *M. catarrhalis *MLST database [66] to identify the sequence variant (SV) number for each gene, as well as the composite sequence type (ST). Phylograms were constructed using the PHYLIP and Phylodendron packages implemented at [67].

### Whole Genome Alignment

Prior to whole genome alignment, contig sequences from each of the eleven draft genome were ordered relative to the closed, completed RH4 chromosome using the contig mover application (Mauve Package v2.3.1; [38]). Pair-wise and multiple genome alignments were performed with the progressiveMauve application (Mauve Package v2.3.1). Both applications were run with their default settings.

### Codon Usage Analysis

Cumulative codon usage was calculated for the total *M. catarrhalis *supragenome and the core and distributed genomes using the GCUA software package (v1.2; [68]). As this analysis is dependent on confident predictions of start sites and in-frame translation, 908 of 21,960 sequences were excluded from GCUA analysis for meeting one or more of the following criteria: 1) presence of internal stop codons, 2) incomplete final codons, 3) unrecognized start codons and/or 4) the absence of synonymous codon usage. The CodonW software package (v1.4; [69]) was used to calculate various codon usage statistics using default settings. In order to prevent sample size-dependent bias during these calculations, one sequence for each of the 2,383 gene clusters was computationally selected without human intervention for further analysis. Of the 2,383 sequences selected, 142 were excluded from these analyses as they fulfilled the exclusion criterion described previously. Inspection of the reduced dataset identified a similar number of sequences from each genome (data not shown).

### Mobile Genetic Element Analysis

Phage and plasmid-derived genes were identified by querying the PGAAP database entry of each genome for phage or plasmid-associated annotation data. A gene was determined to be phage or plasmid-associated if annotations records contained the terms "phage", "plasmid", "transposon" or "Tn".

### CRISPR Analyses

Putative CRISPR elements were identified using CRISPRFinder [70]. CRISPR elements were reported only if CRISPRFinder determined these elements to be of high confidence; possible CRISPR elements were not included in these analyses. High confidence CRISPR elements were subjected to sequence homology analyses using the MacVector package (v11.1.1; MacVector, Inc., Cary, NC) to ascertain homology.

### Metabolic Reconstruction Analysis

Reconstruction of the central metabolic pathways of the RH4 strain was performed as described [13]. Briefly, KEGG orthology (KO) identifiers were assigned with the web-based KEGG automatic annotation server (KAAS; [71]) using the bidirectional best-hit method with the prokaryotic reference set. The central metabolic pathways of all *M. catarrhalis *strains were compared to the RH4 genome [13] using the KO identifiers and PGAAP-based comparative genomics data. For genes that appeared to be missing in the initial PGAAP annotation, the respective genomic regions were examined by additional GLIMMER analysis [72] and manual inspection. Further, for genes that were absent in RH4 [13,73], BlastN searches were performed using coding sequences of bacterial species closely related to *M. catarrhalis*, namely *Psychrobacter *sp., *Acinetobacter *sp., and *Neisseria *sp.

### Statistical Analysis

Pearson correlation analysis was utilized for comparisons of various data sets as denoted in the text. All statistical analyses were performed with Prism v4.0 (GraphPad Software, Inc).

## Authors' contributions

JJD contributed to draft genome assembly and performed genome assembly validation, performed whole genome synteny, codon usage, CRISPR, mobile genetic element and virulence gene conservation analyses, contributed to MLST analysis, and was responsible for data analysis and wrote the manuscript. JE was responsible for genome annotation, supragenome modelling, prepared gene cluster data and performed comparative gene cluster analyses, Neighbour Grouping analysis, contributed to MLST analysis, contributed to data analysis and wrote part of the manuscript. SdV and HJB performed analysis and interpretation of (metabolic) data and wrote part of the manuscript. KS contributed to data analysis. PWMH and HJB supervised work at RUNMC. AA and FZH were responsible for sequencing work performed at CGS. GDE, JPH and AAC were responsible for study design and coordination. All authors critically reviewed the manuscript.

## Supplementary Material

Additional file 1 **Table S1: Reconstruction of M. catarrhalis Central Metabolic Pathways**. Highlighted cells denote differences between strains; n.p. = ORFs not present in one or more strains. * denotes inclusion of RH4 gene annotation data obtained using the Institute for Genome Science annotation engine [13], while ^ denotes RH4 gene annotation data obtained using PGAAP; all other genomes were analyzed with annotation data obtained from PGAAP. Click here for file

Additional file 2 **Table S2: Conservation of known and putative virulence factors**. Each genome was queried for the presence of known or putative *M. catarrhalis *virulence factors by annotation record and/or sequence homology. * Denotes presence of ORF(s) homologous to *mchA1/2 *over 1 Kb or greater of the total length of the ORF. Click here for file
