# Supplementary material for: Comparative analysis and supragenome modeling of twelve Moraxella catarrhalis clinical isolates
Source: BMC Genomics. 2011 Jan 26;12:70. doi: 10.1186/1471-2164-12-70 (PMC3045334; doi:10.1186/1471-2164-12-70)
Supplement: Additional file 2 — Table S2: Conservation of known and putative virulence factors. Each genome was queried for the presence of known or putative M. catarrhalis virulence factors by annotation record and/or sequence homology. * Denotes presence of ORF(s) homologous to mchA1/2 over 1 Kb or greater of the total length of the ORF. [file 1471-2164-12-70-S2.PDF]

Table S2: Conservation of known and putative virulence factors.

Gene	Function	No. Genomes
**Antibiotic, Metal and Reactive Oxygen Species Resistance:**		
MCR_1299	M35-like porin protein	12
MCR_1247	outer membrane porin M35	12
acrA	RND system membrane fusion protein AcrA	12
acrB	RND system efflux pump AcrB	12
ahpC	alkyl hydroperoxide reductase subunit C	12
MCR_0043	AhpD-like alkylhydroperoxidase	12
MCR_0110	Bcr/CflA subfamily drug resistance transporter	12
corC	magnesium and cobalt efflux protein CorC	12
MCR_0808	glyoxalase/bleomycin resistance protein/dioxygenase	12
MCR_1465	putative efflux pump component MtrF	12
MCR_0016	multidrug efflux pump ABC transporter ATPase subunit	12
katA	catalase	12
oprM	RND system membrane channel OprM	12

MCR_0120	putative iron-dependent peroxidase	12
sodA	superoxide dismutase A	12
MCR_1539	transmembrane efflux protein	12
**Iron Acquisition:**		
MCR_0645	NRAMP family Mn2+ and Fe2+ transporter	12
afeA	chelated iron ABC transporter substrate binding protein AfeA	12
afeB	chelated iron ABC transporter ATPase subunit AfeB	12
afeC	chelated iron ABC transporter permease protein AfeC	12
afeD	chelated iron ABC transporter permease protein AfeD	12
fbpA	Iron (III) ABC transporter iron binding protein FbpA	12
fbpB	Iron (III) ABC transporter membrane permease FbpB	12
fbpC	Iron (III) ABC transporter ATPase subunit FbpC	12
MCR_0372	Fur family transcriptional regulator	12
fur	ferric uptake regulation protein Fur	12

copB	outer membrane protein CopB	12
tbpA	transferrin binding protein A TbpA	12
tbpB	transferrin binding protein B TbpB	12
**LOS biosynthesis:**		
kdsA	3-deoxy-8-phosphooctulonate synthase KdsA	12
lgt1	glucosyltransferase Lgt1	12
lgt2A	galactosyltransferase Lgt2A	5
lgt2B/C	galactosyltransferase Lgt2B/C	7
lgt3	glucosyltransferase Lgt3	12
lgt4	acetylglucosaminyltransferase Lgt4	7
lgt5	galactosyltransferase Lgt5	12
lgt6	glucosyltransferase Lgt6	12
**DNA Uptake:**		
comEA	competence protein ComEA	12
comEC	competence protein ComEC	12
comF	competence protein ComF	12
comM	competence protein ComM	12

Adhesins and Outer Membrane Surface Structures:		
hag	*Moraxella* IgD binding protein/hemagglutinin MID/Hag	12
mcaP	*Moraxella catarrhalis* adherence protein McaP	12
*mchA1/A2**	*Moraxella* HMW protein MchA1/A2	8
mchB	*Moraxella* HMW protein tps partner mchB	9
ompCD	outer membrane protein CD	12
ompG1a	outer membrane protein G1a OmpG1a	12
pilA	type IV pilin PilA	12
pilB	type IV pilus assembly ATPase PilB	12
pilC	type IV pilus assembly protein PilC	12
pilD	type IV prepillin peptidase PilD	12
pilMN	type IV pilus biogenesis protein PilMN	12
pilO	type IV pilus biogenesis protein PilO	12
pilP	type IV pilus biogenesis protein PilP	12
pilQ	type IV pilus secretin PilQ	12
pilT	type IV pilus retraction ATPase PilT	12

pilV	type IV pilus biogenesis protein PilV	12
pilW	type IV pilus assembly protein PilW	12
MCR_1173	putative type IV pilus biogenesis protein	12
uspA1	ubiquitous surface protein A1 UspA1	12
uspA2/A2H	ubiquitous surface protein A2/A2H (UspA2/A2H UspA2H)	12
**Environment Sensing:**		
MCR_0179	two component system sensor histidine kinase	12
MCR_0386	two-component system sensor histidine kinase	12
MCR_0387	two-component system sensor histidine kinase	12
MCR_0180	two-component system response regulator	12
MCR_0385	two-component system response regulator	12

* Denotes presence of ORF(s) homologous to *mchA1/2* over 1 Kb or greater of the total length of the ORF.
